# Classification of motor imagery electroencephalogram signals by using adaptive cross-subject transfer learning

**DOI:** 10.3389/fnhum.2022.1068165

**Published:** 2022-12-21

**Authors:** Jin Feng, Yunde Li, Chengliang Jiang, Yu Liu, Mingxin Li, Qinghui Hu

**Affiliations:** ^1^School of Electronic Information and Automation, Guilin University of Aerospace Technology, Guilin, Guangxi, China; ^2^Department of Student Affairs, Guilin Normal College, Guilin, Guangxi, China; ^3^School of Computer Science and Information Security, Guilin University of Electronic Technology, Guilin, Guangxi, China; ^4^School of Computer Science and Engineering, Guilin University of Aerospace Technology, Guilin, Guangxi, China

**Keywords:** brain-computer interface, motor imagery, cross-subject transfer learning, kernel mean matching, TrAdaBoost

## Abstract

**Introduction:**

Electroencephalogram (EEG)-based motor imagery (MI) classification is an important aspect in brain-computer interfaces (BCIs), which bridges between neural system and computer devices decoding brain signals into recognizable machine commands. However, due to the small number of training samples of MI electroencephalogram (MI-EEG) for a single subject and the great individual differences of MI-EEG among different subjects, the generalization and accuracy of the model on the specific MI task may be poor.

**Methods:**

To solve these problems, an adaptive cross-subject transfer learning algorithm is proposed, which is based on kernel mean matching (KMM) and transfer learning adaptive boosting (TrAdaBoost) method. First, the common spatial pattern (CSP) is used to extract the spatial features. Then, in order to make the feature distribution more similar among different subjects, the KMM algorithm is used to compute a sample weight matrix for aligning the mean between source and target domains and reducing distribution differences among different subjects. Finally, the sample weight matrix from KMM is used as the initialization weight of TrAdaBoost, and then TrAdaBoost is used to adaptively select source domain samples that are closer to the target task distribution to assist in building a classification model.

**Results:**

In order to verify the effectiveness and feasibility of the proposed method, the algorithm is applied to BCI Competition IV datasets and in-house datasets. The results show that the average classification accuracy of the proposed method on the public datasets is 89.1%, and the average classification accuracy on the in-house datasets is 80.4%.

**Discussion:**

Compared with the existing methods, the proposed method effectively improves the classification accuracy of MI-EEG signals. At the same time, this paper also applies the proposed algorithm to the in-house dataset, the results verify the effectiveness of the algorithm again, and the results of this study have certain clinical guiding significance for brain rehabilitation.

## 1 Introduction

Brain-computer interface (BCI) (Lin et al., 2021) is a technology, which directly establishes information interaction and control between the brain and external devices. BCI is able to translate neural responses into control instructions by decoding brain activity patterns from electroencephalogram (EEG) (Narayanan and Bertrand, 2019) signals. Motor Imagery (MI) (Tang et al., 2022) based BCI paradigm (MI-BCI) is one of the most popular paradigms nowadays. MI is defined as the cognitive process in which a person imagines their muscles or limbs moving without actually moving. Due to the above characteristics, the MI-BCI system has attracted wide attention in fields as stroke rehabilitation, wheelchair, or prosthetic control, etc. For example, stroke patients can achieve rehabilitation training through MI (Wang et al., 2019) to improve their motor function and restore the ability to control body parts. Therefore, accurate identification and classification prediction of MI-BCI signals is of great significance in the field of medical rehabilitation.

However, generally the number of subjects participating in the experiment is limited, which causes the dataset to be of small sized. In addition, there are great individual differences between subjects and between sessions (Saini et al., 2020), and it is difficult to directly use the MI-EEG signals across subjects to construct a robust classification model. Thus, how to effectively distinguish the types of MI-EEG signals and achieve high performances with small-sized datasets has become the key challenge of MI-BCI system. Transfer learning improves model performance in target tasks by learning and transferring information from source tasks. Some researchers hope to reduce the individual differences in the distribution of MI-EEG signals through transfer learning (Cao et al., 2021; Xu et al., 2021), and build a classification model with high robustness on the MI-EEG signals of all subjects.

Transfer learning mainly includes three learning paradigms: parameter transfer learning, feature transfer learning, and instance transfer learning. For the parameter transfer learning, there is a supervised weighted logistic regression-based transfer learning (S-wLTL), which added the regularization parameters to the objective function of the classifier to make the classification parameters as close as possible to other similar subjects. The classification accuracy of this method on BCIIV dataset IIa reaches 75.6%, which is higher than that of common spatial pattern (CSP) combined with support vector machine (SVM) (Azab et al., 2019). Based on the MI-EEG signals of other source subjects, a parallel multiscale filter bank convolutional neural network is pre-trained, and then fine-tuned in the individual target task. The experimental results show that the classification accuracy of cross-subject classification reaches 75.9% (Wu et al., 2019). However, when the parameters of the source domain are transferred to the target domain, the catastrophic forgetting problem may occur with the iterative optimization of the algorithm, which leads to low accuracy of the results obtained by these methods (Guo et al., 2021).

For the feature transfer learning, the algorithm based on the maximum mean difference (MMD) (Pan et al., 2010) is the common method. MMD is used to select features that are more relevant to the target task by measuring the distribution difference between source and target domain samples. Li et al. (2020) proposed a discriminative transfer feature learning (DTFL), which enhances class discrimination information by minimizing the marginal and condition distribution between the source and target domain, while maximizing the distance between the classes. The experimental results show that classification accuracy is 83.5%. In addition, a weighted logistic regression method (Chen et al., 2022) based on Euclidean feature space is proposed, and the MI-EEG signals of different subjects are aligned in Euclidean space (He and Wu, 2019) to reduce the difference of signals. Then the Kullback–Leibler (KL) divergence (Shibanoki et al., 2018) of CSP features between different subjects is calculated in Euclidean space, and the average classification accuracy of this method is 85%. The feature transfer learning method reduces the distribution differences between the source and target domain by specific data distribution analysis methods. However, when the data distribution between source and target domain samples are very different, the effect of feature transfer learning will be seriously affected, and even may cause negative transfer (Wan et al., 2021).

For the instance transfer learning, the main idea is to train the target classifier by selecting the source domain samples with more similar distribution to the target samples. For examples, Tan et al. (2018) proposes an instance transfer ensemble learning for Alzheimer’s disease classification, which is used to select and transfer source domain samples with similar to target domain samples by instance transfer learning algorithm (ITL) based on wrapper mode, thereby obtaining optimal transferred domain samples. Dai et al. (2007) proposed a boost transfer learning (TrAdaBoost) algorithm, which applied the idea of adaptive boosting (Adaboost) (Chen et al., 2021) in transfer learning to improve the instance weight of the target classification task and reduce the instance weight of the unfavorable target task. Similarly, Huang et al. (2007) proposed a kernel mean matching (KMM) algorithm, which aims to make the probability distribution of the weighted source domain sample and the target domain sample as close as possible.

Inspired by the above studies, this study combines KMM and TrAdaBoost algorithms to analyze MI-EEG signals for the first time and proposes a KMM-TrAdaBoost instance transfer learning algorithm. The KMM-TrAdaBoost is mainly used to solve the problem that the small number of training samples of MI-EEG for a single subject and the great individual differences of MI-EEG signals among different subject resulting the poor performance of the classification model. Firstly, the algorithm preprocesses the source and target domain samples to extract their CSP features. Then uses the KMM algorithm to obtain the sample weight matrix between source and target domain, which is used as the initial weight matrix of TrAdaBoost to solve the problem that TrAdaBoost algorithm is sensitive to the initialization weight. Finally, the strong classifier, which integrates multiple weak classifiers based on TrAdaBoost is used for classification. The KMM-TrAdaBoost algorithm proposed in this paper can effectively improve the classification accuracy for a single subject in the case of small samples. At the same time, the model also showed good performance on the samples of different subjects, and obtained high classification accuracy and generalization performance, which reduced the influence of individual differences on MI-EEG classification. In addition, this method has certain clinical value for cross-individual rehabilitation therapy.

## 2 Methodology

### 2.1 Overview

**FIGURE 1:**
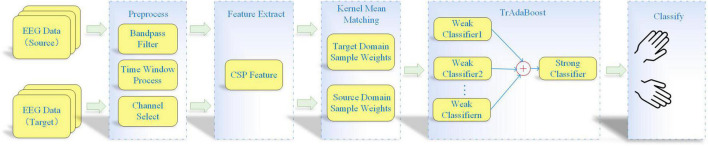
Flow chart of kernel mean matching-transfer learning adaptive boosting (KMM-TrAdaBoost) algorithm.

The flow chart of proposed KMM-TrAdaBoost is shown in Figure 1. Firstly, the KMM algorithm is used to calculate the weights matrix of all training sample to estimate the similarity between the source and target sample. Then the matrix is used as the initialization weight matrix of TrAdaBoost algorithm. Finally, the strong classifier, which integrates multiple weak classifiers based on TrAdaBoost is used for classification. The specific algorithm steps are as follows:

(1) **Preprocessing all source and target domain data**. The preprocessing steps comprise band-pass filtering, time window processing, and channel selection.
(2) **Spatial features extracting**. Spatial features are extracted from the preprocessed data by using CSP algorithm.
(3) **Calculating sample weight matrix**. The KMM algorithm is used to obtain a sample weight matrix, which makes the mean values of the features between the source and target domain as close as possible.
(4) **Training several weak classifiers**. The sample weight matrix, which is calculated by KMM algorithm, is as an initialization sample weight matrix of the TrAdaBoost algorithm. Then, several weak classifiers are trained based on the weighted samples.
(5) **Obtaining strong classifier by voting strategy**. According to the weight of each classifier, the final strong classifier is obtained by using the voting strategy.

### 2.2 CSP algorithm

**FIGURE 2:**
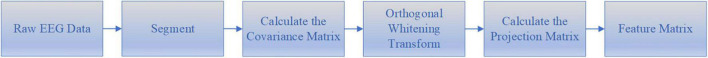
Flow chart of common spatial pattern (CSP) algorithm.

Common spatial pattern (Ang et al., 2012) is a spatial filtering feature extraction algorithm for two-class MI-EEG tasks, which aims to use matrix diagonalization to find a set of optimal spatial filters and project them to maximize the variance difference between the two class of signals. And then, the specificity feature vectors with high discrimination were obtained to achieve the task of distinguishing two kinds of MI-EEG signal. The CSP algorithm flow is shown in Figure 2.

Suppose *X*_*1*_ and *X*_*2*_ are the time-space signal matrices of multichannel evoked responses under the dichotomous MI task, and their dimensions are N*T. *N* are the number of channels of the EEG device, and *T* is the number of sampling points of each channel. *X*_*1*_ and *X*_*2*_ can be expressed as:


(1) $$X_{1}=\left[\begin{array}{cc} C_{1} & C_{M} \end{array}\right]\,\left[\begin{array}{c} S_{1}\\ S_{M} \end{array}\right]\;X_{2}=\left[\begin{array}{cc} C_{2} & C_{M} \end{array}\right]\,\left[\begin{array}{c} S_{2}\\ S_{M} \end{array}\right]$$

Where *S*_*1*_ and *S*_*2*_ represents two classification MI tasks in source domain, and they are assumed to be linearly independent of each other. *S*_*M*_ represents a source signal that is common to both types of tasks, assuming that *S*_*1*_ consists of *m*_*1*_ source domain signals and *S*_*2*_ consists of *m*_*2*_ source domain signals. Then, *C*_*1*_ and *C*_*2*_ are composed of *m*_*1*_ and *m*_*2*_ common spatial patterns associated with *S*_*1*_ and *S*_*2*_, respectively. Each spatial pattern is a N*1 dimensional vector, which represents the distribution weight of the signal caused by a single source signal on *N* leads. *C*_*M*_ represents the corresponding shared spatial pattern with *S*_*M*_. The goal of CSP algorithm is to design a spatial filter to obtain the spatial factor *W*, and its process is shown as follows. (1) Normalizing *X*_*1*_ and *X*_*2*_, respectively, their corresponding covariance matrices *R*_*1*_ and *R*_*2*_ are calculate. They can be expressed as:


(2) $$R_{1}=\frac{X_{1}\,X_{1}^{T}}{t\,r\,a\,c\,e\,\left(X_{1}\,X_{1}^{T}\right)}\;R_{2}=\frac{X_{2}\,X_{2}^{T}}{t\,r\,a\,c\,e\,\left(X_{2}\,X_{2}^{T}\right)}\,$$

Where *trace*(•) is the sum of the entries on the diagonal for the matrix, and *T* is the transpose of the matrix. (2) The mixed spatial covariance *R* from *R*_*1*_ and *R*_*2*_ can be expressed as:


(3) $$R=\bar{R_{1}}+\bar{R_{2}}$$

Where $\bar{R_{1}}$ and $\bar{R_{2}}$ are the average covariance matrices, respectively.

Based on the eigenvalue decomposition theory, *R* is expressed as *R* = *U*λ*U*^*T*^, where *U* is an eigenvector of the matrix, λ is the corresponding eigenvalue. In addition, the eigenvalues are arranged in descending order, and the corresponding eigenvectors are also rearranged. Then, principal component analysis theory (Ahuja et al., 2022) was used to calculate the whitening matrix_*P*_, and it be expressed as:


(4) $$P=\sqrt{\lambda^{-1}}\,U^{T}$$

(3) Based on the whitening matrix_*P*_, the covariance matrix *R*_*1*_ and *R*_*2*_ can be transformation, and it is expressed as:


(5) $$S_{1}=P\,R_{1}\,P^{T}\;S_{2}=P\,R_{2}\,P^{T}$$

Where *S*_*1*_ and *S*_*2*_ have a common eigenvector. *if S*_1_ = *B*λ_1_*B*^*T*^, then *S*_2_ = *B*λ_2_*B*^*T*^ and λ_1_ + λ_2_ = *I*, where *B* is common eigenvector of *S*_*1*_ and *S*_*2*_, and *I* is the identity matrix. Since the eigenvalues of the two matrices always add up to 1, the eigenvector of *S*_*1*_ corresponding to the largest eigenvalue cause *S*_*2*_ to have the smallest eigenvalue.

The projection matrix *W* is the corresponding spatial filter, and it be expressed as *W* = *B*^*T*^*P*. Then the EEG data of the single task experiment *X*_*i*_ can be transformed into *Z* = W*X_*i*_*i* = (1,2). For the EEG signals, the feature value *f*_*p*_ can be expressed as:


(6) $$f_{p}=\mathrm{lg}\left[\frac{v\,a\,r\,\left(Z_{p}\right)}{s\,u\,m\,\left(v\,a\,r\,\left(Z_{p}\right)\right)}\right]\;p=\left(1,2,3\,\ldots ,2\,m\right)$$

### 2.3 KMM-TrAdaBoost algorithm

#### 2.3.1 KMM algorithm

Instance transfer learning is to select samples from the source domain, which are consistent with the distribution of the target samples, and improves model performance in target tasks by transferring information from source domain. The KMM (Huang et al., 2007) algorithm maps the source domain samples from original feature space into reproducing kernel Hilbert space (RKHS) (Gertton et al., 2012), and then calculates the difference between the mean value of the source and target domain data under the RKHS space. Finally, a set of weight parameters matrix are obtained, which are used to weight the samples in the source domain to make the probability distribution consistent with the samples in the target domain. The calculation process of KMM algorithm is as follows.


(7) $$\min\,{||\frac{1}{m}\,\sum_{i=1}^{m}\beta_{i}\,\phi \,\left(x_{i}^{s}\right)-\frac{1}{n}\,\sum_{i=1}^{n}\phi \,\left(x_{i}^{t}\right)||}_{H}^{2}$$

Where $x_{i}^{s}$ is a set of source domain sample (*i* = 1,2,…,*m*), and$x_{i}^{t}$is a set of target domain sample (*i* = 1,2,…,*n*). H denotes the RKHS with a characteristic kernel *k*. β_*i*_ ∈ [0,1] represents the weight of the *i*−*th* source domain sample. ϕ(•) is the mapping function from the original space to the RKHS, and satisfies the following relation: < ϕ(*x*), ϕ(*y*) >_*H*_ = *k*(*x*, *y*). *k*(*x*,*y*) is a Gaussian kernel function, namely:


(8) $$k\,\left(x,y\right)=\exp\left(-{||x-y||}^{2}\,/\,2\,\sigma^{2}\right)$$

where σ represents the size of the Gaussian kernel. Combining Equations 7, 8, the MMD between each source and target domain is defined as:


(9) $$\min\left(\frac{1}{m^{2}}\,{\left(\sum_{i=1}^{m}\beta_{i}\,\phi \,\left(x_{i}^{s}\right)\right)}^{2}-\frac{2}{n\,m}\,\sum_{i=1}^{n}\beta_{i}\,\phi \,\left(x_{i}^{s}\right)\,\sum_{i=1}^{n}\phi \,\left(x_{i}^{t}\right)+c\right)$$

where *c* stands for constant, and $\sum_{i=1}^{m}\beta_{i}\,\phi \,{\left(x_{i}^{s}\right)}^{2}$ and $\sum_{i=1}^{n}\beta_{i}\,\phi \,\left(x_{i}^{s}\right)\,\sum_{i=1}^{n}\phi \,\left(x_{i}^{t}\right)$ can be reduced to a matrix form as:


(10) $$\begin{array}{c} {\left(\sum_{i=1}^{m}\beta_{i}\,\phi \,\left(x_{i}^{s}\right)\right)}^{2}\\ ={\left(\beta_{1}\,\phi \,\left(x_{1}^{s}\right)+\ldots +\beta_{m}\,\phi \,\left(x_{n}^{s}\right)\right)}^{2}\\ ={\left[\beta_{1}\,\beta_{2}\,\ldots \,\,\beta_{m}\right]}^{*}\,{[\begin{array}{ccc} k\,\left(x_{1}^{s},x_{2}^{s}\right) & \ldots & k\,\left(x_{1}^{s},x_{m}^{s}\right)\\ \begin{array}{c} .\\ . \end{array} & & \begin{array}{c} .\\ . \end{array}\\ k\,\left(x_{m}^{s},x_{1}^{s}\right) & \ldots & k\,\left(x_{m}^{s},x_{m}^{s}\right) \end{array}]*}^{\;}{\left[\beta_{1}\,\,\beta_{2}\,\ldots \,\,\beta_{m}\right]}^{T}=\beta^{T}\,K \end{array}$$

(11) $$\begin{array}{c} \sum_{i=1}^{m}\beta_{i}\,\phi \,\left(x_{i}^{s}\right)\,\sum_{i=1}^{n}\phi \,\left(x_{i}^{t}\right)\\ ={[\sum_{j=1}^{n}k\,\left(x_{1}^{s},x_{j}^{t}\right)\,\,\ldots \,\,\sum_{j=1}^{n}k\,\left(x_{n}^{s},x_{j}^{t}\right)]*}^{\;}\,{\left[\beta_{1}\,\,\beta_{2}\,\,\ldots \,\,\beta_{m}\right]}^{T}=g^{T}\,\beta \end{array}$$

Where $K_{i,j}=k\,\left(x_{i}^{s},x_{j}^{s}\right)$ and $g_{j}=\frac{m}{n}\,\sum_{j=1}^{n}k\,\left(x_{i}^{s},x_{j}^{t}\right)$. Then the final quadratic optimization objective function is as follows.


(12) $$\min\left(\frac{1}{n^{2}}\,\beta^{T}\,K\,\beta -\frac{1}{m^{2}}\,g^{T}\,\beta \right)$$

#### 2.3.2 TrAdaBoost algorithm

**FIGURE 3:**
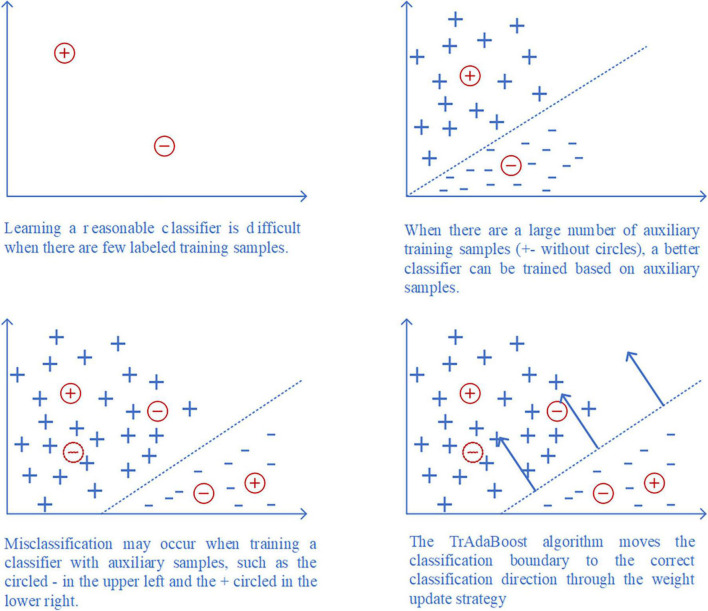
Flow chart of TrAdaBoost algorithm.

Inspired by the algorithm of AdaBoost (Chen et al., 2021), TrAdaBoost (Dai et al., 2007) uses weight automatic updating mechanism to constantly adjust the weight of samples, so as to keep important source domain samples and eliminate the samples that are not similar to the distribution of target domain samples. The principle of the TrAdaBoost algorithm is shown in Figure 3.

$D^{s}={\left\{\left(x_{i}^{s},y_{i}^{s}\right)\right\}}_{i=1}^{n}$ represents samples from source domain, where $x_{i}^{s}\in D^{s}\left(i=1,\ldots ,n\right)$ is a sample example, $y_{i}^{s}$ is the corresponding ground-truth labels. $D^{t}={\left\{\left(x_{j}^{t},y_{j}^{t}\right)\right\}}_{j=1}^{m}$ represents samples from target domain, where $x_{j}^{t}\in D^{t}\left(j=1,\ldots ,m\right)$ is a sample example,$y_{j}^{t}$ is the corresponding ground-truth labels. *n* and *m* represent the sizes of *D*^*s*^ and *D*^*t*^. *T*^*t*^ is the test samples from target domain, which is assumed to follow a different probability distribution from *D*_*s*_ and the same probability distribution as *D*_*t*_. The goal of transfer learning is to train a classifier*c*with the minimum classification error based on a small amount of target domain samples *D*_*t*_ and a large amount of source domain samples *D*_*s*_. The specific algorithm flow is shown as follows.

(1) Initialize samples weight $w^{1}=\left(w_{1}^{1},\ldots ,w_{n+m}^{1}\right)$ from source and target domain samples *D* = {*D*^*s*^, *D*^*t*^}:


(13) $$w_{k}^{1}=\frac{1}{n+m},k=1,\ldots ,n+m$$

(2) Set initial parameter β of source domain sample:


(14) $$\beta =1/\left(1+\sqrt{2\,\ln n/N}\right)$$

Where *N* is the number of iterations.

(3) Normalizing the weight vector *w*, and a weak classifier *h*_*l*_ is trained based on the weighted sample *D* and test data *T*^*t*^, and SVM is adopted as a weak classifier in this paper. Calculate the error of the weak classifier *h*_*l*_ on the training dataset of the target domain, and the calculation formula is as follows.


(15) $$\varepsilon_{l}=\sum_{i=n+1}^{n+m}\frac{w_{i}^{l}|h_{l}\left(x_{i}^{t}\right)-y_{i}^{t})|}{\sum_{i=n+1}^{n+m}w_{i}^{l}}$$

Where *l* = 1,…,*N* represents the *l*−*th* iteration.

(4) Set up β_*l*_ = ε_*l*_/(1−ε_*l*_), and update weight


(16) $$w_{i}^{l+1}=\left\{ \begin{array}{c} w_{i}^{l}\,\beta^{\left|h_{l}\left(x_{i}\right)-y_{i}^{t})\right|}\;i=1,2,3,\ldots ,n\\ w_{i}^{l}\,\beta_{l}^{-|h_{l}\left(x_{i}\right)-y_{i}^{t})|}\,i=n+1,\ldots ,n+m \end{array}\right.$$

(5) Repeat steps (3–4) and iterate for *N* times to obtain the final strong classifier


(17) $$h\,\left(x\right)=\left\{ \begin{array}{c} 1,\sum_{l=\left\lceil N/2\right\rceil }^{N}\ln\left(1/\beta_{l}\right)\,h_{l}\,\left(x\right)\geq \frac{1}{2}\,\sum_{l=\left\lceil N/2\right\rceil }^{N}\ln\left(1/\beta_{l}\right)\\ 0,\mathrm{other} \end{array}\right.$$

### 2.4 Statistical analysis for different datasets

For different datasets, the two-tailed *t*-test (Xie et al., 2022) was used to compare the significant difference between the proposed method and state-of-the-art methods, and *P* < 0.05 was considered statistically significant. All statistical tests were carried out with Origin 2022 software.

## 3 Result and discussion

### 3.1 Dataset

#### 3.1.1 Public dataset

**FIGURE 4:**
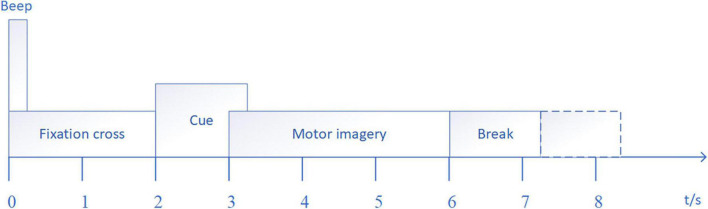
Experimental paradigm.

**FIGURE 5:**
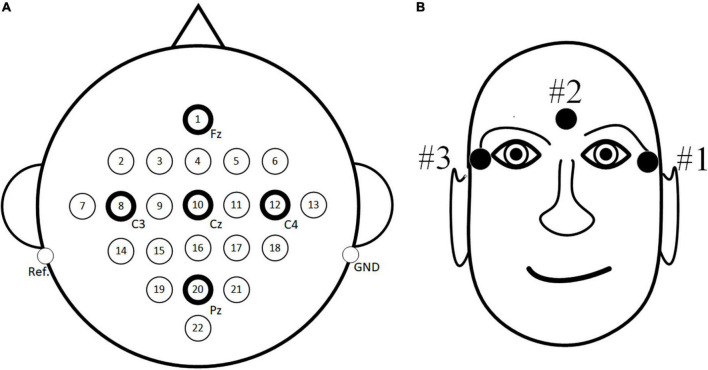
The position of electrode. **(A)** Electroencephalogram (EEG) electrode position **(B)** EOG electrode position.

For the public dataset 1, the BCI Competition IV dataset 2a (Blankertz et al., 2006) is used for research. The dataset consisted of EEG signals recorded by 22 electrodes from nine subjects with three EOG scalp electrode locations. The dataset consists of four tasks, which are left hand, right hand, foot and tongue MI task. 72 MI experiments were performed for each task, and a total of 288 experiments. The signal sampling frequency was 250 Hz, and the band pass filter between 0.5–100 Hz and notch filter of 50 Hz are used to eliminate the power frequency interference. In order to be consistent with the in-house dataset, only the MI data of the left and right hands MI task are used in this paper. Specific experimental paradigms are shown in Figure 4, and EEG electrode positions are shown in Figure 5A as follows: Fz, FC3, FC1, FCz, FC2, FC4, C5, C3, C1, Cz, C2, C4, C6, CP3, CP1, CPz, CP2, CP4, P1, Pz, P2, and POz, left mastoid reference, right mastoid grounding. The position of EOG electrode is shown in Figure 5B.

For the public dataset 2, BCI Competition IV dataset I (Blankertz et al., 2006) is adopted for research. The data set was EEG signals recorded by 59 electrodes in 7 subjects. There are three categories of MI tasks, namely left and right hand and foot imagination. The sampling frequency is 100 Hz. In order to ensure the consistency of experimental paradigm, left and right hand categories are extracted from three motor imagination tasks in this paper, and each category of each user has 100 samples. Electrode position adopts international 10/20 system.

#### 3.1.2 In-house dataset

An in-house dataset was recorded from 20 subjects (S01-S20) who are divided into two groups, including ten (S01-S10) who are good at table tennis and 10 (S11-S20) who are not. Scan software, Neuroscan37 Quik-Cap electrode cap and SynAmps2 amplifier, and MATLAB software were used for data acquisition and analysis. The prompting mode of “preparation-prompt-movement” was adopted. The prompting was divided into two ways: imaginary right-handed attack and left-handed attack. Each player did 144 experiments. The dataset collected in this paper consists of 32 electrodes, A1 and A2 are used as reference electrodes, and EEG signals are collected from the scalp of each subject at a sampling frequency of 250 Hz. “HEOL” and “HEOR”’ is used to obtain the horizontal electro-oculogram. “VEOU” and “VEOL” is used to obtain the vertical electro-oculogram.

### 3.2 Data preprocessing

In the data preprocessing stage, 3 EOG channels were deleted and 22 EEG channels are retained for the public dataset. Different from the public dataset, the in-house dataset deletes the 6 electrode data of “HEOL,” “HEOR,” “A1,” “A2,” “VEOU,” and “VEOL.” 26 EEG channels are retained to reduces the false recording caused by the EOG signal. According to the principle of event related desynchronization (ERD) and event related synchronization (ERS) (Tang et al., 2022), when people perform MI tasks, the cerebral cortex will produce obvious rhythm signals, which are divided into 8–12 Hz μ rhythm signals and 13–30 Hz β rhythm signals. In order to improve the signal-to-noise ratio of EEG signals, 8–30 Hz band-pass filtering is used to process the data and remove the baseline. At the same time, the corresponding EEG signal was extracted as the subsequent analysis data by using the time window of 0.5–3.5 s after the appearance of the prompt.

The MI-EEG data used in this paper consists of two parts, including nine subjects from the IV2a dataset (A01, A02,…, A09) and twenty subjects (S01-S20) from the in-house dataset. For the IV2a dataset, the EEG data of one subject was selected as the target domain data, and the data of the other eight subjects was selected as the source domain data. For the 20 subjects from the in-house dataset, they were divided into two groups: 10 subjects who could play ping-pong and 10 subjects who could not. There were 10 subjects in each group. One subject from the same group was selected as the target domain data, and the remaining 9 subjects were used as the source domain data. In each set of data, 70% of the data is the training set and 30% of the data is the test set.

### 3.3 Result evaluation index

To evaluate the performance of the classification model, the accuracy (Acc) and Kappa value (K) are measured, and the calculation equations are as follows:


(18) $$\left\{ \begin{array}{c} A\,c\,c=\frac{T\,P+T\,N}{T\,P+F\,N+T\,N+F\,P}\\ K=\frac{A\,c\,c-p_{e}}{1-p_{e}} \end{array}\right.$$

Where TP is the number of samples correctly classified as positive label, TN is the number of samples correctly classified as negative label, FP is the number of samples misclassified as positive label, and FN is the number of samples misclassified classified as negative label. *P*_e_ = (*a*_1_×*b*_1_ + *a*_2_×*b*_2_ + … + *a*_*z*_×*b*_*z*_)/*n*^2^ represents the random classification rate of model to samples. *a*_1_,*a*_2_,…,*a*_*z*_ represents the actual sample size of each type of sample, and *b*_1_,*b*_2_,…,*b*_*z*_ represents the number of samples of each type predicted by the model *n* is the total number of samples. Through the comparative analysis of Kappa values, the influence of random classification on the accuracy of the model can be eliminated.

### 3.4 Analysis of experimental results

#### 3.4.1 Comparison with state-of-the-art methods

In order to verify the effectiveness of the proposed algorithm (Ours), the following two methods are compared, including the EA-CSP-LDA (He and Wu, 2019) and CA-JDA (Iturrate et al., 2013) algorithm.

The EA-CSP-LDA algorithm reduces the difference of cross-subject signals based on the Euclidean space data alignment approach. For the CA-JDA algorithm, joint distribution adaptation (JDA) is used to align the edge probability distribution and conditional probability distribution of cross-subject signals to achieve effective transfer learning. From these results in Table 1, we can obtain the following insightful observations.

**TABLE 1:** Results of comparison with state-of-the-art methods on the public dataset 1 and public dataset 2.

	Acc				K		
	Method	EA-CSP-LDA	CA-JDA	Ours	EA-CSP-LDA	CA-JDA	Ours
Public dataset 1	A01	86.2%	65.8%	**92.5%**	0.72	0.43	**0.81**
	A02	58.6%	51.8%	**89.7%**	0.43	0.35	**0.75**
	A03	96.6%	65.9%	**92.6%**	0.90	0.44	**0.83**
	A04	71.6%	62.3%	**81.9%**	0.57	0.46	**0.65**
	A05	54.6%	54.8%	**90.2%**	0.36	0.40	**0.79**
	A06	66.9%	58.6%	**85.6%**	0.46	0.42	**0.69**
	A07	68.9%	67.2%	**91.7%**	0.48	0.47	**0.78**
	A08	86.9%	88.2%	**89.9%**	0.73	0.70	**0.71**
	A09	77.9%	71.9%	**87.9%**	0.60	0.58	**0.76**
	Average ± Std	(74.2 ± 12.3)%	(65.1 ± 9.5)%	**(89.1 ±** 3.1)%	0.58 ± 0.16	0.47 ± 0.09	**0.75 ±** 0.05
Public dataset 2	A01	75.8%	62.8%	**89.5%**	0.59	0.46	**0.75**
	A02	75.9%	59.9%	**83.5%**	0.59	0.39	**0.67**
	A03	74.8%	60.9%	**84.6%**	0.58	0.40	**0.69**
	A04	73.5%	61.3%	**85.1%**	0.56	0.41	**0.70**
	A05	90.6%	68.6%	**90.8%**	0.79	0.48	**0.81**
	A06	77.8%	67.8%	**80.9%**	0.61	0.47	**0.60**
	A07	85.9%	61.8%	**84.2%**	0.71	0.41	**0.62**
	Average ± Std	(79.1 ± 5.6)%	(63.3 ± 3.0)%	**(85.5 ±** 2.9)%	0.63 ± 0.07	0.43 ± 0.03	**0.69 ±** 0.06

Std stands for standard deviation. Bold indicates the maximum value in the current indicator.

(1) Compared with the proposed method, the EA-CSP-LDA and CA-JDA algorithms do not take into account the samples with large differences, especially for the recognition of cross-individual MI-EEG signals, so the performance of the above algorithms is poor.

(2) The proposed method obtains the best prediction results on the MI-EEG classification tasks, verifying its effectiveness and superiority.

#### 3.4.2 Ablation experiment

To verify the effectiveness of the proposed algorithm (Ours), ablation experiments are carried out for the following model: CSP+KMM, CSP+TrAdaboost, and CSP+SVM.

The ablation experiment is carried out on public dataset 1 and public dataset 2, and the results are shown in Table 2. It can be seen from Table 2 that the average classification accuracy of CSP+KMM algorithm is only 84.1%. The main reason is that KMM is an unsupervised algorithm and cannot effectively use the label information of source and target domain data. The average classification accuracy of CSP+TrAdaboost algorithm is only 77.9%, the main reason is that TrAdaboost is sensitive to the sample initialization weight matrix, while the traditional TrAdaboost algorithm assigns the same initialization weight to all samples. Compared with the traditional TrAdaboost algorithm, this paper uses the KMM algorithm to calculate the importance of samples as the initial sample weight matrix of TrAdaboost. Experimental results verify the effectiveness of this idea, and the average classification accuracy of the proposed algorithm is 89.1%, which is higher than that of other classification methods. The average classification accuracy of the CSP+SVM algorithm is only 58.5%, which proves that there is a big difference between the data of each subject in MI task, and the model trained by other subjects cannot be well used for the current subject.

**TABLE 2:** Comparison of the accuracies (%) between the proposed algorithm and other methods on the public dataset 1 and public dataset 2.

	Target	CSP+ KMM	CSP+ TrAdaboost	CSP+ SVM	Ours
Public dataset 1	A01	83.3%	75%	60.7%	**92.5%**
	A02	87.3%	78.4%	59.6%	**89.7%**
	A03	86%	76.8%	63.5%	**92.6%**
	A04	78.4%	72.3%	53.9%	**81.9%**
	A05	85.6%	80.4%	54.6%	**90.2%**
	A06	78.3%	72.6%	67.2%	**85.6%**
	A07	87.8%	84.3%	54.3%	**91.7%**
	A08	85.6%	81.4%	58.2%	**89.9%**
	A09	84.2%	80.3%	54.9%	**87.9%**
	Average ± Std	(84.1 ± 3.1)%	(77.9 ± 3.6)%	(58.5 ± 4.1)%	**(89.1 ±** 3.1)**%**
Public dataset 2	A01	73.3%	78.6%	60.4%	**89.5%**
	A02	77.3%	79.4%	57.6%	**83.5%**
	A03	76.5%	78.8%	53.5%	**84.6%**
	A04	74.4%	76.7%	58.9%	**85.1%**
	A05	72.6%	80.4%	54.9%	**90.8%**
	A06	74.3%	72.8%	58.2%	**80.9%**
	A07	72.8%	84.3%	54.3%	**84.2%**
	Average ± Std	(74.4 ± 1.5)%	(78.7 ± 3.0)%	(56.8 ± 2.2)%	**(85.5 ±** 2.9)**%**

Std stands for standard deviation. Bold indicates the maximum value in the current indicator.

#### 3.4.3 Algorithm robustness analysis

**FIGURE 6:**
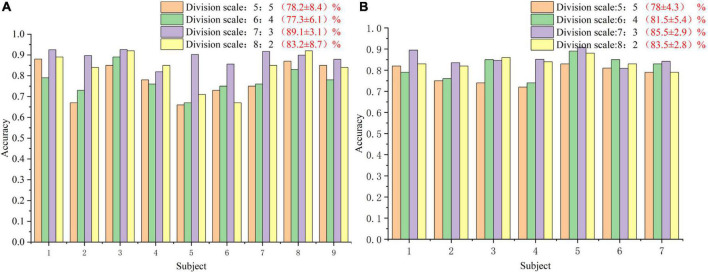
The classification accuracy curve at different training sizes. **(A)** The result of public dataset 1. **(B)** The result of public dataset 2.

In order to analyze the robustness of the our algorithm, different numbers of training trials were analyzed, including five groups of 5:5, 6:4, 7:3, and 8:2, respectively. The results are shown in Figure 6. For different groups, the algorithm presented in this paper shows good classification accuracy and robustness, with the average accuracy of 82.0% and the highest average accuracy of 82.1% in the public dataset 1 and 2, respectively. For the groups of 5:5 and 6:4, the classification performance of the algorithm is relatively poor because there are too few training samples. In view of the above problems, we plan to carry out further research on the classification algorithm of motion imagination signals under small samples in the future. In addition, In order to measure the statistical significance of our algorithm, we conducted a *T*-test statistical analysis for the above two state-of-the-art algorithms and the algorithm in this paper. The results are shown in Tables 3–5.

**TABLE 3:** *T*-test results for the proposed method vs. state-of-the-art methods.

Paired *T*-test	*P*-value	
	Public dataset 1	Public dataset 2
Ours vs. EA-CSP-LDA	*	*
Ours vs. CA-JDA	*	**

∼ nonsignificant, *P ≤ 0.05, **P ≤ 0.01.

**TABLE 4:** Separate session of *T*-test statistical analysis for the proposed method vs. state-of-the-art methods.

Paired *T*-test	*P*-value	
	Ours vs. EA-CSP-LDA	Ours vs. CA-JDA
A01	*	**
A02	*	*
A03	∼	**
A04	*	*
A05	*	*
A06	*	*
A07	*	*
A08	∼	∼
A09	*	*
Average	*	*

∼ nonsignificant, *P ≤ 0.05, **P ≤ 0.01.

**TABLE 5:** Separate session of *T*-test statistical analysis for the proposed method vs. state-of-the-art methods.

Paired *T*-test	*P*-value	
	Ours vs. EA-CSP-LDA	Ours vs. CA-JDA
A01	*	**
A02	∼	**
A03	*	*
A04	*	*
A05	∼	*
A06	*	**
A07	∼	**
Average	*	*

∼ nonsignificant, *P ≤ 0.05, **P ≤ 0.01.

#### 3.4.4 Application of algorithm on the in-house dataset

**FIGURE 7:**
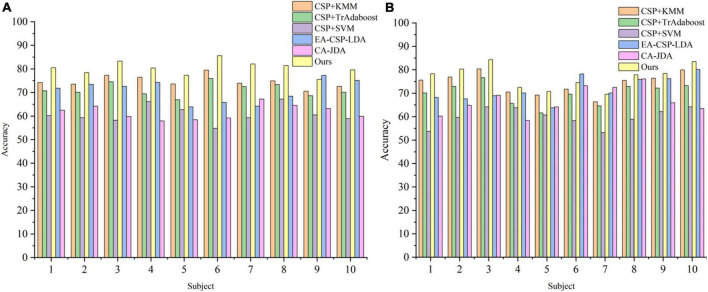
The classification accuracy curve in the in-house dataset. **(A)** The result of table tennis group. **(B)** The result of non-table tennis group.

To verify the superiority of the proposed method, a comparative experiment is carried out on in-house dataset, and the results are shown in Figures 7A, B. For the local dataset, our algorithm still outperforms the other three comparison algorithms.

**FIGURE 8:**
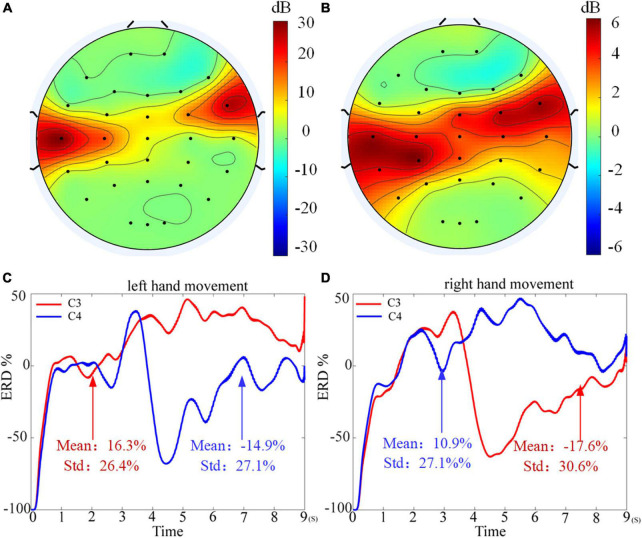
Topographic map of weight distribution. **(A)** The result of table tennis group. **(B)** The result of non-table tennis group. **(C)** The event related desynchronization (ERD) analysis for the left hand motor imagination. **(D)** The ERD analysis for the right hand motor imagination.

Meanwhile, by comparing the average classification accuracy between the table tennis group and the non-table tennis group, it can be seen that the average classification accuracy obtained by each algorithm of the table tennis group is better than that of the non-table tennis group. Previous studies have shown that when athletes repeatedly train the same skills for a long time, the number of neural synapses in the brain will change and the connections between various brain regions will be strengthened (Ericsson and Lehmann, 1997). Correspondingly, it will also enhance the activation degree of the motor center, which can help athletes to complete training more quickly and stably (Scott, 2004). In order to show whether the corresponding brain region will have corresponding responses during motor imagination, the topographic map are drawn in the Figure 8 (see Supplementary Appendix 1 for drawing details). For the two groups of subjects, it can be seen that the response area is at the regions corresponding to the primary motor cortex. From the color bar on the right, the non-table tennis group has a relatively weak degree of activation, while the participants who could play table tennis has a relatively strong degree of activation. Further, for the ERD analysis (see Supplementary Appendix 2 for drawing details), Figure 8C shows that the right areas (C4) shows a power decrease in specific frequency bands when the left hand movement is imagined, while Figure 8D shows that the left areas (C3) shows a power decrease in specific frequency bands when the right hand movement is imagined. Based on the above theory, we can try to carry out rehabilitation training for stroke patients for a certain period of time, which has certain clinical guiding significance for brain rehabilitation treatment.

## 4 Conclusion

Facing the small number of MI-EEG training samples of a single subject and the large individual differences of MI-EEG signals among different subjects, an instance transfer learning algorithm based on KMM-Tradaboost was proposed. In this paper, the algorithm firstly preprocesses the source and target domain data, and then extracts the spatial features of the preprocessed data by using the CSP algorithm. After obtaining the spatial features, the KMM algorithm is used to calculate the sample weight matrix and initialize the TrAdaBoost algorithm. Finally, a strong classifier is trained by the TrAdaBoost algorithm after the initial weights are assigned, which is used to classify the MI-BCI data. The experimental results show that the algorithm proposed in this paper is superior to other algorithms, and provides a new idea to solve above problems. At the same time, this paper also applies the proposed algorithm to the in-house dataset, the results verify the effectiveness of the algorithm again, and the results of this study have certain clinical guiding significance for brain rehabilitation.

## Data availability statement

The original contributions presented in this study are included in the article/Supplementary material, further inquiries can be directed to the corresponding author.

## Ethics statement

Ethical review and approval was not required for the study on human participants in accordance with the local legislation and institutional requirements. Written informed consent for participation was not required for this study in accordance with the national legislation and the institutional requirements.

## Author contributions

JF and YLiu designed the research. JF wrote the manuscript. CJ and ML collected the data and supervised the study. YLi and QH supervised and revised the manuscript. All authors contributed to the article and approved the submitted version.
