# Supplementary material for: Classification of motor imagery electroencephalogram signals by using adaptive cross-subject transfer learning
Source: Front Hum Neurosci. 2022 Dec 21;16:1068165. doi: 10.3389/fnhum.2022.1068165 (PMC9811670; doi:10.3389/fnhum.2022.1068165)
Supplement: Supplementary file 1 [file Data_Sheet_1.docx]

**Supplement material**

**Supplementary A1: Brain topographic map mapping process**

**Firstly**, the data was preprocessed according to Section 3.2 (***Data preprocessing***) of the paper.

**Second**, power spectral density (PSD) was extracted for each channel according to Wiener-Khintchine method [1]. The specific calculation process is as follows.

①The preprocessed data of the channel $x=[x_{1},x_{2},...,x_{i},...,x_{L}]$ is divided into $L$ segments,and each segment of data $x$ contains $M$ sampling points.

②In order to facilitate signal processing, this study uses the Hamming window function to add windows to each section of signal

③The power spectral is calculated by using the fast Fourier transform (FFT) based on the above data，as shown below

$p(e^{-j\omega})=\frac{1}{MU}\left|\sum_{n=1}^{M} x_{i}(n)w(n)e^{-j\omega n}\right|^{2}$ (1)

Where $w(n)$ is the Hamming window function, and $x_{i}(n)$represents the $n\text{-th}$ sampling point of the $i\text{-th}$signal $x_{i}$. $U=\frac{1}{M}\sum_{\text{n}=1}^{M} \omega^{2}(n)$ is the normalization factor, which is mainly used to ensure that the results obtained by spectral estimation are asymptotic and unbiased.

④The power spectral density is obtained by summing and averaging the estimated power spectrum from $L$ segments.

$p(e^{-j\omega})=\frac{1}{MUL}\sum_{\text{i}=1}^{L} \left|\sum_{n=1}^{M} x_{i}(n)w(n)e^{-j\omega n}\right|^{2}$ (2)

**Third**，Since the EEG signals collected only come from a limited number of fixed collection points on the subject's head, the blank Spaces between each point need to be filled with some interpolation formula, and the power ratio method for baseline correction [2].

**Finally**, according to electrode position and power spectrum value, color mapping of power spectrum value is carried out, and the power spectrum value is mapped to the corresponding electrode position of scalp, so as to obtain the final Brain topographic map.

**Supplementary A2:The principle of Event Related Desynchronization (ERD) and Event Related Synchronization (ERS)**

According to the principle of Event Related Desynchronization (ERD) and Event Related Synchronization (ERS), when people perform MI tasks, the cerebral cortex will produce obvious rhythm signals [3]. The specific calculation process is as follows.

(1)First, a specific frequency band is selected(according to reference [4], alpha band is a more important frequency band), and each channel Electroencephalogram (EEG) data is performed using a bandpass digital filter. In this paper, Butterworth filter is used for digital filtering.

(2)After each channel Electroencephalogram (EEG) data is performed using a Butterworth filter, the mean value $\overline{x}_{j}$of all EEG samples was calculated.

(3)Each sample value is subtracted from the above mean and the difference squared operation is performed as shown in the following formula

$\text{y}_{ij}=(x_{ij}-\overline{x}_{j})^{2}$ (3)

Where $x_{ij}$ is the $j\text{-th}$ sampled value of the $i\text{-th}$ test after filtering, and $\overline{x}_{j}$ is the mean of all the $j\text{-th}$ sampled values after filtering.

(4)The power sampling mean is obtained by calculating the mean of the difference squared, the specific formula is as follows

$A_{j}=\frac{1}{N-1}\sum_{i=1}^{N} y_{ij}$ (4)

(5)Average several consecutive data values and reduce the original sampling rate to a reasonable empirical sampling rate.

(6)A certain period before the event is selected as the reference interval, and the average energy is calculated with the following formula.

$R=\frac{1}{k}\sum_{r_{0}}^{r_{0}+k} A_{j}$ (5)

Where $R$ is the average energy in the interreference period $[r_{0},r_{0}+k]$, and $r_{0}$is the beginning point of the interreference period, $k$ is the length of the interreference period.

(7)Finally, A decrease or increase in the percentage of energy is calculated by the following formula.

$ERD_{j}=\frac{A_{j}-R}{R}*100\%$ (6)

**Reference**

[1] Yu, Y. and Zhao, H. (2012). A new method for noise power spectrum estimation. 4th IET International Conference on Wireless, Mobile and Multimedia Networks (ICWMMN) 206-209. doi:10.1049/cp.2011.0990

[2] Roach, B., J., and Mathalon, D., H. (2008). Event-related EEG time-frequency analysis:An overview of measures and an analysis of early gamma band phase locking in schizophrenia. Schizophrenia Bulletin 34(5),907-926. doi: 10.1093/schbul/sbn093.

[3] Graimann, B., Huggins, J., Levine, S., and Pfurtscheller, G. (2002)Visualization of significant ERD/ERS patterns in multichannel EEG and ECoG data. Clinical Neurophysiology 113(1):43-47. doi:10.1016/S1388-2457(01)00697-6.

[4] Krause, C., Sillanmaki, L., Haggqvist, A., and Heino, R. (2001).Test-retest consistency of the event-related desynchronization/event-related synchronization of the 4-6, 6-8, 8-10 and 10-12 Hz frequency bands during a memory task. Clinical Neurophysiology 112(5).750-757. doi:10.1016/S1388-2457(01)00501-6.
